# Multiple Ganglion Cysts of the Wrist and Leg in a 70-Year-Old Female: A Case Report

**DOI:** 10.7759/cureus.103598

**Published:** 2026-02-14

**Authors:** Ramachandra Reddy Gowda Venkatesha, Karthik Rajaram Mohan, Nandhini Chandran, Saramma Mathew Fenn, R.T.Reethika Rathan

**Affiliations:** 1 Oral Medicine and Radiology, Vinayaka Mission's Sankarachariyar Dental College, Vinayaka Mission's Research Foundation (Deemed to be University), Salem, IND

**Keywords:** arthroscopic surgery, aspiration hazard, hyaluronidase, sodium tetradecyl sulfate, volar wrist ganglion cyst

## Abstract

A ganglion cyst is a noncancerous, fluid-filled swelling that usually forms near joints or tendons, with the wrist and hand being the most common locations. These cysts can range in size from small, pea-sized bumps to larger formations similar to the size of a golf ball, and their texture may vary from firm to spongy. Smaller cysts can be asymptomatic and are usually round or spherical in shape. The skin over the cyst may appear stretched and shiny. Ganglion cysts contain a mucoid, gel-like material rich in mucopolysaccharides. Some cysts occur intratendinously, extending deep into the underlying muscle, while others may extend into the carpal bone, causing pain in the wrist or leg. Ganglion cysts that are asymptomatic and do not cause pain typically do not require treatment. This case highlights the occurrence of asymptomatic ganglion cysts in the wrist and leg of a 70-year-old woman, discussing their etiology, theories of formation, clinical features, differential diagnosis, and treatment.

## Introduction

A ganglion cyst is a fluid-filled sac that typically forms near joints or tendons, most commonly in the hand or wrist [1]. These cysts can range in size from as small as a pea to about an inch in diameter and are generally round or oval in shape [1]. The fluid inside is typically a thick, jelly-like substance that resembles synovial fluid, which lubricates the joints [1]. Ganglion cysts contain hyaluronic acid, along with smaller amounts of glucosamine, globulin, and albumin [1]. The majority of ganglion cysts (70-80%) occur on the wrist, with the most common locations being the dorsal (posterior) side near the scapholunate ligament and the volar (anterior) side near the radioscaphoid joint [1]. They can also appear in various places on the fingers, such as at the distal interphalangeal joint (tip joint) or below the cuticle, where they are referred to as mucous cysts [1,2]. Approximately 11% of ganglion cysts occur on the foot or ankle, often due to conditions such as arthritis or bone spurs [1,2]. Ganglion cysts may also form around the elbow, though this is less common [2,3]. In rare cases, they occur in the shoulder area, particularly in the spinoglenoid and suprascapular notches [1-3].

## Case presentation

A 70-year-old woman presented to our Department of Oral Medicine for a routine dental checkup. Her medical history revealed that she had been experiencing joint pain in the knee for the past 15 years. She had no known comorbidities such as hypertension or diabetes, and there was no family history of similar swellings near the wrist or other joints. She reported no history of trauma to her wrist or ankle joints. The swelling in her wrist and knee joints had been present for approximately one year. The swellings in her wrist and ankle joints were entirely asymptomatic. She denied any deleterious habits such as chewing or smoking tobacco, betel nut use, or alcohol consumption.

**Figure 1:**
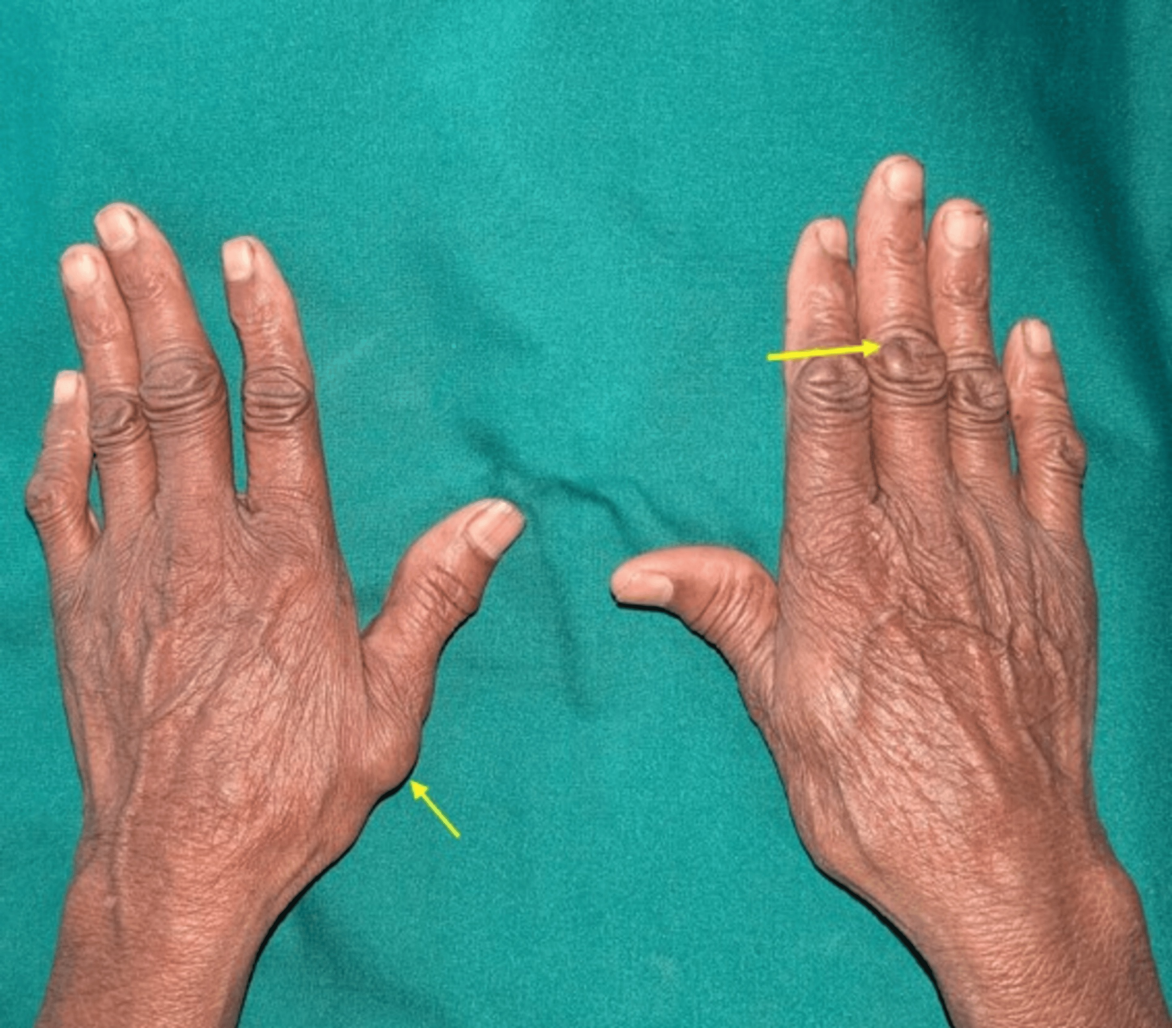
Examination of the wrist revealed swellings near the left thumb and the third interphalangeal joint (yellow arrow)

On general examination, her height was 170 cm, weight 60 kg, blood pressure 129/76 mmHg, and pulse rate 83 beats per minute. Clinical examination of the extensor aspect of her wrist revealed a swelling near the right wrist joint, located at the volar aspect of her right arm (Figure 1).

**Figure 2:**
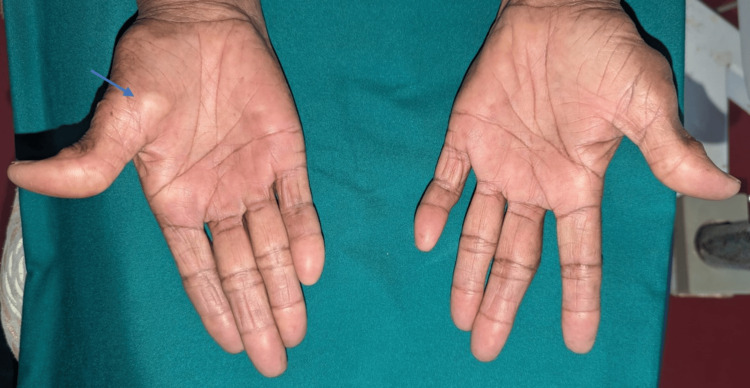
Examination of the flexor aspect of the hand revealed swelling at the base of the right thumb (blue arrow)

Examination of the flexor aspect revealed a swelling approximately 1 cm in diameter, firm in consistency, and nontender on palpation. The transillumination test was positive. The skin over the swelling appeared stretched and shiny (Figure 2).

**Figure 3:**
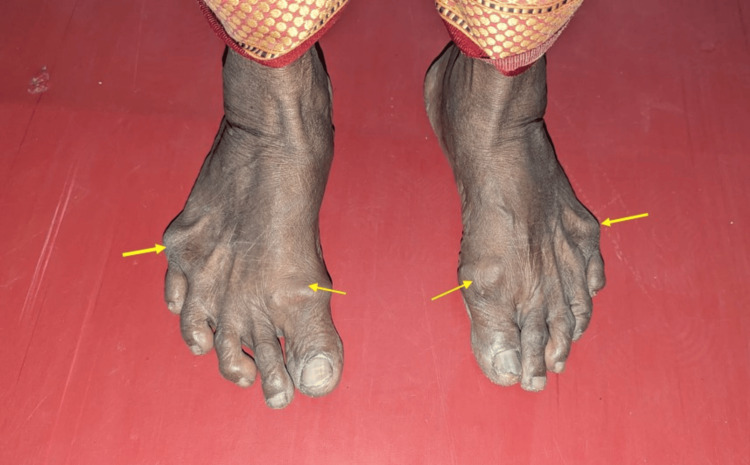
Examination of the leg revealed swellings near the right and left great and small toes (yellow arrow)

Examination of her leg revealed swellings near the right and left great and small toes. The transillumination test was positive. Each swelling measured approximately 1 cm in diameter, was fluctuant, and nontender (Figure 3).

The clinical differential diagnosis included Heberden nodes, Bouchard nodes, and rheumatoid nodules. Heberden nodes, seen in osteoarthritis, occur at the distal interphalangeal joints and are typically asymptomatic, nontender, and bony-hard in consistency. Bouchard nodes, also associated with osteoarthritis, appear at the proximal interphalangeal joints. Rheumatoid nodules, which are firm and often tender, occur around the joints and elbows in advanced cases of rheumatoid arthritis.

Early morning stiffness is a symptom present in both rheumatoid arthritis and osteoarthritis. In osteoarthritis, the stiffness usually subsides within minutes, whereas in rheumatoid arthritis, it tends to persist for hours. Notably, this early morning stiffness does not occur in patients with ganglion cysts.

**Figure 4:**
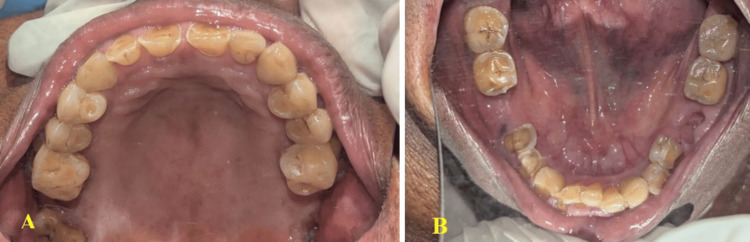
(A) Intraoral examination revealed generalized attrition and a missing maxillary second molar. (B) Missing right and left mandibular first molars.

Intraoral examination revealed generalized attrition of all teeth, along with missing left and right mandibular first molars (Figure 4A, 4B).

**Figure 5:**
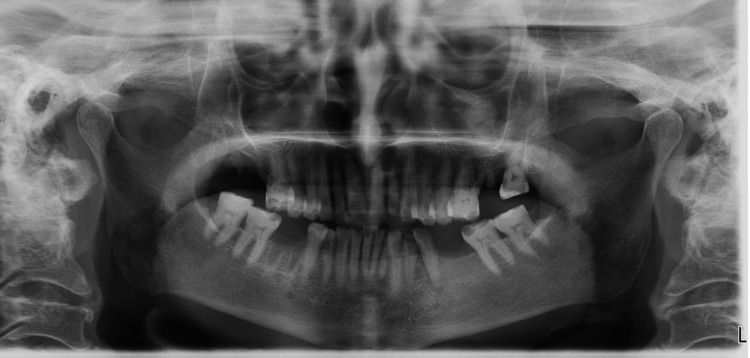
Orthopantomography revealed generalized attrition and missing right and left mandibular first molars, as well as the right maxillary second molar

Orthopantomography revealed missing right and left mandibular first molars and the right maxillary second molar (Figure 5).

Unless ganglion cysts in the wrists and legs cause discomfort, such as pain, or impair joint movement, treatment is generally not required [3]. Management strategies for wrist and leg ganglion cysts depend on several factors, including the size and location of the cyst, whether it is dorsal or volar, the presence of clinical symptoms, and its impact on daily activities. Typically, nonsurgical options are preferred as the initial approach due to their lower risk of complications [3].

Nonsurgical treatment options - such as observation, use of a neoprene wrist brace or splint, compression bandages, custom orthotics, and aspiration - are considered both safe and effective [3]. Aspiration, which involves draining the cyst’s fluid under local anesthesia, is a commonly used method, especially for dorsal-type wrist ganglion cysts [3]. However, aspiration is not recommended for volar-type cysts due to the risk of injury to the radial artery, which could compromise blood circulation [3]. In some cases, intralesional steroid injections may follow aspiration to reduce inflammation and improve outcomes [3]. Nevertheless, Grégoire and Guigal reported that corticosteroid injections do not significantly improve outcomes for dorsal wrist ganglion cysts, aside from minor functional score improvements compared to no treatment [4].

Hyaluronidase, an enzyme that breaks down hyaluronic acid, a key component of the cyst’s gelatinous content, can facilitate aspiration by liquefying the cyst material and increasing the permeability of the cyst wall to steroid injections [5]. If nonsurgical options fail or symptoms persist, surgical excision, known as ganglionectomy or gangliectomy, may be performed via open or arthroscopic methods under local or general anesthesia [5]. Recovery following surgical excision typically ranges from two to six weeks and carries a higher risk of complications, including infection, neuropraxia, numbness, weakness, tingling, hematoma, scarring, neuroma formation, joint stiffness, or reduced grip strength [5].

Ganglion cysts may recur regardless of the chosen treatment modality [6]. Surgical excision generally offers a lower recurrence rate than aspiration [6]. In cases of recurrence following open surgery, arthroscopic excision may be indicated [6]. This minimally invasive procedure uses an arthroscope and small keyhole incisions to enhance visualization of the cyst and its stalk, which is typically attached to a tendon sheath or ligament [6]. The technique enables precise dissection and removal, leading to faster recovery and less postoperative scarring compared to open surgery [6]. Dry arthroscopy, which relies on traction to create working space without fluid irrigation, offers improved visibility using tools such as synoviotomes and neurosurgical patties to keep the field clear [6]. In this case, treatment was not indicated as the patient remained entirely asymptomatic [6].

The modified thread technique is another minimally invasive outpatient method involving the insertion of two sterile silk or linen threads through the cyst wall in a cross pattern to induce inflammation and promote obliteration of the cyst [7]. A sterile gauze is tied to the threads to facilitate removal after seven days [7]. This technique does not require hospitalization and has been reported to be safe and effective, with minimal complications, making it a promising alternative for patients seeking a less invasive option than surgery [7]. Nonetheless, patients should be informed that recurrence is possible regardless of the treatment approach [7].

**Figure 6:**
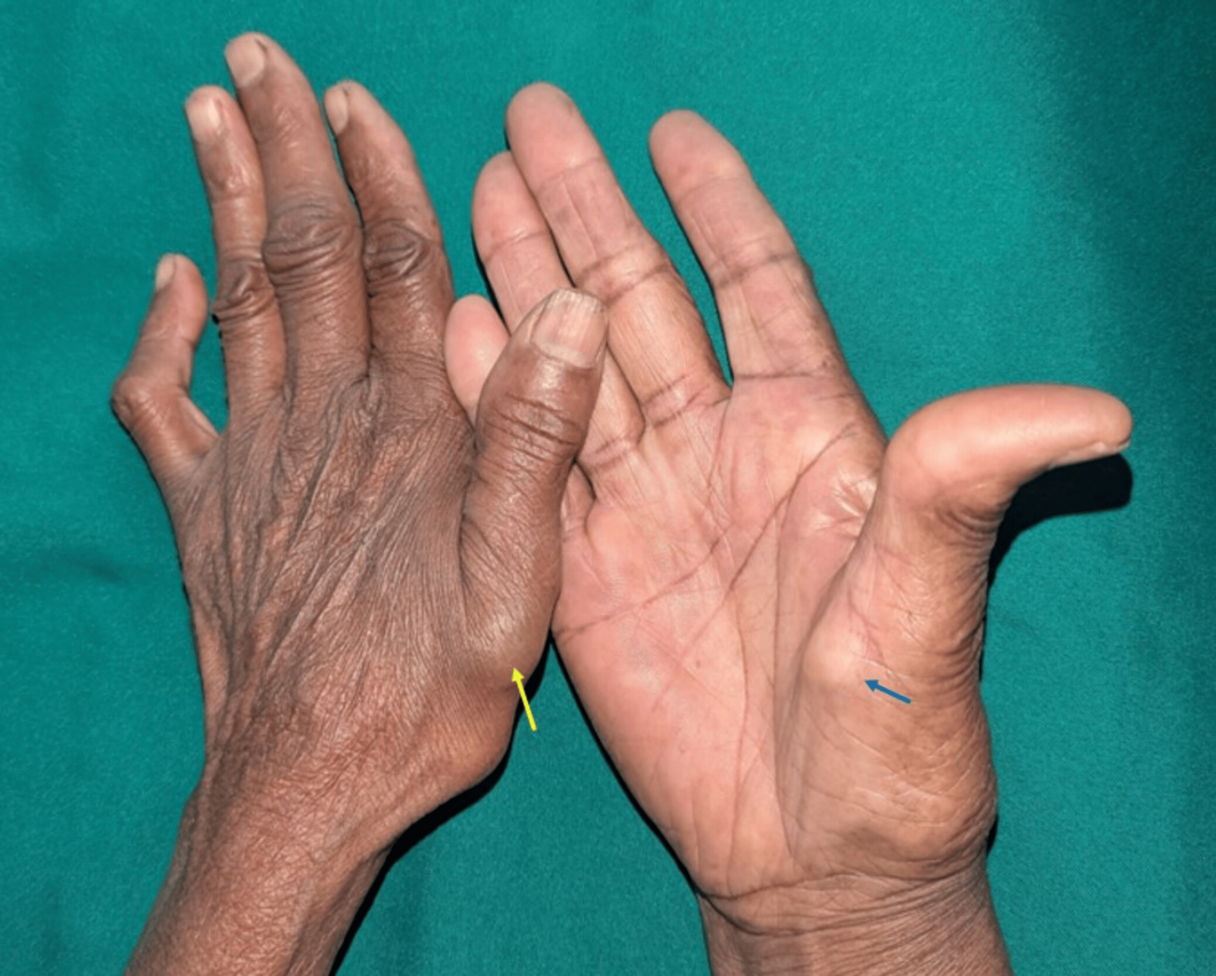
A one-month follow-up examination revealed a dorsal wrist ganglion cyst (yellow arrow) and a volar ganglion cyst (blue arrow)

Regular follow-up is essential to monitor healing and detect any early complications. At a one-month follow-up, the patient reported no worsening of the wrist or leg swellings and remained completely asymptomatic (Figure 6).

Because ganglion cysts on the wrist are benign and effective treatment options exist, the overall prognosis is favorable. However, setting appropriate patient expectations and selecting suitable treatment approaches requires an understanding of the potential for both spontaneous remission and recurrence.

## Discussion

Causes and risk factors

The exact etiology of ganglion cysts is not fully understood, but they are believed to occur when synovial fluid escapes from a tendon sheath or joint due to trauma or repetitive strain [8]. Risk factors for developing ganglion cysts include a history of injury to the wrist or fingers, repetitive wrist motion, and arthritis [8]. Ganglion cysts are more commonly found in patients with osteoarthritis [8].

Theories of the formation of ganglion cysts

The pathophysiology of ganglion cysts is subject to multiple theories, highlighting the complexity of their development. The following are the principal theories of their formation:

Microtrauma and Mucin Synthesis

The most widely accepted theory is that cyclic microtrauma to the adjacent tissue or joint induces mucin production by fibroblasts. The mucin precipitates and forms coalescent droplets that aggregate to become cysts. Stretching the joint capsule and ligaments also induces mucin production, leading to cyst formation [8].

Capsular Rent Theory

Another hypothesis suggests that joint stress, such as peri-scaphoid ligament injury, may cause a tear in the joint capsule, allowing synovial fluid to leak into peri-articular tissues. This leakage can result in a reaction between the fluid and surrounding tissues, ultimately leading to cyst formation. It is believed that joint pathology before the injury may predispose patients to this injury [8].

Myxoid Degeneration

A third theory proposes that chronic trauma or stress to the connective tissue can lead to myxoid degeneration, in which the connective tissue degenerates and fluid accumulates, forming a cyst. This is similar to the mucin production hypothesis but emphasizes degenerative changes rather than direct trauma [8].

Clinical presentation

Ganglion cysts typically present as smooth swellings under the skin, filled with a thick, jelly-like substance resembling synovial fluid, which lubricates joints [9]. These cysts may be visible or palpable and can change size over time. While most ganglion cysts are asymptomatic and require no treatment, they can cause discomfort or pain if they irritate surrounding nerves or restrict joint movement [9]. Common symptoms include a palpable mass on the wrist, hand, ankle, or foot, pain or discomfort, particularly with joint movement, and tingling or numbness if the cyst compresses a nerve. The size of the cyst may vary, and some cysts resolve spontaneously without any treatment [9]. A dorsal wrist ganglion is typically hard, spherical, and painless, although compression of surrounding terminal branches of the posterior interosseous nerve may cause pain [9]. Treatment options include observation, needle aspiration, or surgical removal [9].

Diagnosis and treatment

Diagnosis is typically made through history and physical examination [9]. MRI is recommended only for occult wrist ganglion cysts [9]. Surgical excision of the dorsal wrist ganglion cyst may be performed through either an arthroscopic or open approach [9]. Transillumination helps differentiate cysts from tumors [9]. Most ganglion cysts do not require treatment unless they become painful or cause functional impairment [9]. Treatment options include observation, as many cysts resolve spontaneously, and aspiration, which involves draining the cyst’s fluid using a needle [9]. Surgical treatment may be necessary for volar wrist ganglion cysts in patients at risk for radial artery atherosclerosis [9]. Post-surgical immobilization for a dorsal wrist ganglion cyst typically lasts for two weeks [10].

Furthermore, excision of the radial artery, followed by repair using an autogenous vein, may be a viable surgical option if the ganglion and radial artery are not thoroughly dissected [11]. Surgical excision is indicated if conservative treatments fail [11]. Superficial angiomyxoma in the wrist can sometimes be mistaken for a ganglion cyst [12]. Although ganglion cysts are benign and may spontaneously regress in many cases, symptomatic cysts can be troublesome [12]. Synovial sarcoma is a rare but possible diagnosis in the wrist joint, particularly in a 69-year-old Caucasian woman [13]. Approximately 50% of ganglion cysts may resolve without treatment, but this process can take several months to a few years [14]. Intratendinous ganglion cysts can extend into the muscle belly of the flexor carpi radialis [14]. When symptomatic or troublesome, intervention such as aspiration or surgery can alleviate discomfort and reduce the likelihood of recurrence [14]. Intratendinous ganglion cysts may also extend into the muscle belly of the extensor pollicis longus tendon [14,15]. Intraosseous ganglion cysts within the carpal bones can lead to chronic wrist pain [16]. Chemicals such as hyaluronidase, sodium tetradecyl sulfate, triamcinolone, and hydrocortisone are used in the treatment of ganglion cysts [17]. Ganglion cysts are also referred to as “Bible bumps” [18].

Wrist braces and splints can be effective in managing ganglion cysts by providing support and immobilizing the affected area [19]. A wrist brace is a flexible, functional device that provides support while allowing some movement, typically used for mild cases [19]. A splint, which is a stiffer device, is more commonly prescribed for severe cases [19]. Wrapping the wrist with a compression bandage can provide additional support and reduce swelling associated with the cyst [19]. Both wrist braces and splints help limit joint movement, reduce pressure on the cyst, and alleviate discomfort [19]. Immobilization may also promote healing and potentially reduce the cyst’s size over time [19].

## Conclusions

Ganglion cysts are benign, fluid-filled masses that commonly form around joints or tendon sheaths, most often on the wrist and hand. Although most ganglion cysts are harmless and may resolve spontaneously, treatment is necessary only for symptomatic cysts that cause pain or impair joint function. Aspiration is usually the first-line treatment, where the cyst’s fluid is drained, sometimes with a steroid injection to reduce inflammation. Surgical excision remains the gold standard for treating recurring or symptomatic ganglion cysts, effectively removing the cyst and its stalk to prevent recurrence. Despite their clinical presentation, ganglion cysts rarely cause permanent disability.
